# Topic classification of synchrotron experiment proposals using the OpenAlex model: enhancing metadata granularity and assessing live service feasibility

**DOI:** 10.1107/S1600577526006685

**Published:** 2026-08-03

**Authors:** Terence Tan, Oliver J. Clark, Matthew J. Derry, Renaud Duyme, Guilherme Abreu Faria, Robert Farla, Ralf Flaig, Yogal Prasad Ghimirey, Anushka Ghosh, Miguel A. Gomez-Gonzalez, Ellen L. Heeley, Anna Herlihy, Annette Kleppe, Paul Millar, Melanie Nentwich, Josh Pickston, Nick Terrill, Armin Wagner, Andrew C. Walters, Matthew Watson, Philippe Rocca-Serra, Susanna-Assunta Sansone, Stephen P. Collins

**Affiliations:** a Diamond Light Source Didcot OxfordshireOX11 0DE United Kingdom; b Oxford e-Research Centre, Department of Engineering Science University of Oxford OxfordOX1 3QG United Kingdom; c Aston Institute for Membrane Excellence Aston University BirminghamB4 7ET United Kingdom; d European Synchrotron Radiation Facility 38043Grenoble France; e Helmholtz-Zentrum Hereon, Institute of Materials Physics, 21502Geesthacht, Germany; f Deutsches Elektronen-Synchrotron DESY Notkestr. 85 22607Hamburg Germany; g Research Complex at Harwell Rutherford Appleton Laboratory Harwell DidcotOX11 0FA United Kingdom; h Department of Physics University of Warwick CoventryCV4 7AL United Kingdom; i Department of Biology Humboldt-Universität zu Berlin 10099Berlin Germany; j School of Life, Health and Chemical Sciences The Open University Milton KeynesMK7 6AA United Kingdom; k School of Science, Engineering and Environment University of Salford SalfordM5 4WT United Kingdom; Bhabha Atomic Research Centre, India

**Keywords:** synchrotron experiment proposals, topic classification, OpenAlex, machine learning, FAIR principles

## Abstract

Experiment proposals at synchrotron facilities serve as the primary gateway for instrument access. They currently lack the standardized and granular topic metadata necessary for tasks such as classification and review, and, broadly speaking, reuse. This paper defines and tests the feasibility of a real-time topic classification service for experiment proposals using an open-source machine-learning model and domain experts for the evaluation phase. We applied the OpenAlex topic classification model to 5384 experiment proposals and selected 209 of them to each be independently evaluated by three domain experts to assess the performance and utility of the model. Analysis of the evaluations reveals a general consensus among the reviewers regarding the model’s predictions, with a Krippendorff’s alpha of 0.572. We also find that 74.2% of the proposals had at least one topic that was unanimously deemed relevant, which suggests that the model performs well enough to be used in a live setting with real-time verification. However, we do not recommend using it in automated environments without human oversight, given the proposal-based precision score of 56.0%. By aligning the data infrastructure of photon and neutron facilities with the OpenAlex ecosystem, we also lay the groundwork for the eventual inclusion of proposals and experiment reports into OpenAlex, which is necessary for a complete record of a research activity.

## Abstract 2

This paper validates the OpenAlex model for real-time topic classification of synchrotron proposals. The proposed live service architecture incorporates user verification, providing a scalable solution for introducing enhanced topic metadata into scientific workflows without sacrificing precision.

## 1. Introduction

Synchrotron facilities are an integral component of the worldwide scientific ecosystem, contributing to many areas of scientific research, from palaeontology (Cau *et al.*, 2017 ▸) to drug development (Douangamath *et al.*, 2020 ▸).

While the academic focus has traditionally centred on the scholarly publications and datasets coming out of these facilities, two other types of documents are also a crucial part of synchrotron facilities’ science lifecycle: namely the experiment proposals and experiment reports. The experiment proposal is a document written by a research group to request access to a synchrotron facility instrument for an experiment. It undergoes rigorous peer review by domain experts to determine facility access. The experiment report summarizes the findings and technical outcomes immediately following the conclusion of an experiment.

Currently, scholarly publications and, to a lesser extent, datasets have well developed ecosystems built around them, from DOI minting to representation within bibliographic databases such as Web of Science and Elsevier’s Scopus. In contrast, experiment proposals and reports are notably behind in terms of data infrastructure, confined exclusively to the originating facilities. Worse still, open access to these documents is not guaranteed across all institutions. This lack of discoverability and accessibility is a serious impediment, as these documents are essential for understanding the end-to-end research process. Hence, like other scholarly outputs, they should be made as FAIR (Wilkinson *et al.*, 2016 ▸) as possible.

This paper seeks to contribute to the FAIRification of these overlooked documents by focusing on a specific piece of work that also addresses the immediate needs of the photon and neutron community: the use of machine learning for automatic topic classification of experiment proposals, along with evaluation of the results. Utilizing the OpenAlex classification scheme and model (developed and used by the OpenAlex team), we demonstrate how this approach generates enhanced metadata that could be used for a variety of downstream tasks, such as reviewer-proposal assignment and scientific trend analysis. The validation of the model is an immediate need for the European Synchrotron Radiation Facility (ESRF), which plans to integrate it into a live service.

For simplicity, the term ‘proposals’ is used throughout to refer collectively to both the experiment proposals and the reports, as the latter is a direct extension of the former.

### 1.1. The challenge

Within the facilities’ own repositories, proposals are classified under broad research areas. For the ESRF, these research areas come in the form of twelve Scientific Disciplines: CH – Chemistry; ES – Earth Science; EV – Environment; HC – Hard Condensed Matter Science; HG – Cultural Heritage; LS – Life Sciences; MA – Applied Material Science; MD – Medicine; ME – Engineering; MI – Methods and Instrumentation; MX – Structural Biology/Macromolecular Crystallography; SC – Soft Condensed Matter Science. These internal classifications of proposals are broad and do not conform to existing metadata standards. This is also true for other facilities like Diamond Light Source, which classifies its scientific activities into nine similar Research Areas: Chemistry; Earth Sciences and Environment; Engineering and Technology; Life Sciences; Materials Science; Physics; Energy; Cultural Heritage; COVID-19.

The low granularity reduces the utility of current metadata for downstream tasks, such as tracking specific trends over time. The absence of standardization also results in a lack of semantic interoperability across facilities. This effectively creates isolated data silos, significantly impeding the discovery, integration, and analysis of proposal data by the scientific community, and making cross-referencing with external databases, such as OpenAlex, unnecessarily complex.

In addition, facilities like ESRF could introduce a live service that will ingest a submitted proposal and output topic predictions for the beam time applicant to select in real time. The performance of the underlying machine-learning model must be rigorously evaluated prior to its official deployment.

These two factors, the development and validation of the live service and the concurrent need to enhance the granularity and standardization of proposal topic metadata, converge to form the central challenge that this paper addresses.

### 1.2. The solution

Our work uses machine learning to augment the existing broad internal classifications used by ESRF with more granular, standardized topics already used by OpenAlex. OpenAlex is chosen because it is open-source, non-proprietary, and has a huge coverage of different scholarly outputs and research domains (see Section 2.1 for a more detailed discussion). Additionally, the OpenAlex team has already developed their own topic classification model (OpenAlex, 2024*a* ▸) for journal articles (see Section 2.4), allowing us to repurpose it for ESRF experiment proposals (see Section 3.2) to enhance proposal topic metadata, as mentioned above in Section 1.1. The outputs of the model were then evaluated by domain experts to assess the model’s performance (Section 3.3), followed by a comprehensive analysis of the results (Section 4).

ESRF documents were chosen because, although the proposals are not fully publicly available, the experiment reports are easily accessible and can serve as a suitable proxy for input to the model (see Section 3.1). This approach satisfies publication requirements by ensuring that all input data are openly accessible. Furthermore, proposal documents are similar in structure (with the same key components such as title, abstract, and references *etc*.) across most photon and neutron facilities, making the findings of this study broadly applicable to other sites, including Diamond Light Source.

The results of this study will also serve as validation for a live service (Section 5.1) that ESRF is considering implementing. This service will provide real-time topic predictions for beam-time applicants to select directly on the ESRF User Portal during proposal submission.

## 2. Background

### 2.1. Bibliographic databases

Bibliographic databases are digital catalogues of references to published sources, especially journal articles and conference proceedings, which are indexed with additional metadata such as titles, abstracts, and author names (Gasparyan *et al.*, 2013 ▸).

The Web of Science and Scopus are perhaps two of the most well known bibliographic databases. However, they are subscription-based services, which poses a barrier to open science. In contrast, OpenAlex is free to use and has better coverage of scholarly outputs, particularly those in Diamond open access journals (Simard *et al.*, 2024 ▸).

Dimensions is an alternative database that was considered. A study found that Dimensions had much better coverage of academic journals than Web of Science and Scopus (Singh *et al.*, 2021 ▸). Like OpenAlex, it also classifies topics at the article level. Dimensions’ classification system for topics, referred to as Fields of Research (FoR), is a component of the 2020 Australian and New Zealand Standard Research Classification (ANZSRC) system (Australian Bureau of Statistics, 2020 ▸). It has three hierarchical levels (from broadest to most granular): Divisions, Groups, and Fields. The developers of Dimensions trained a machine-learning model for FoR classification at the broader Divisions and Groups level (Porter *et al.*, 2023 ▸).

In contrast, OpenAlex’s system of classification works on a much more granular level (see Section 2.3), making it the more attractive option for this project. In addition, OpenAlex has received support from major organizations. The Centre for Science and Technology Studies (CWTS) at Leiden University in the Netherlands decided to base the CWTS Leiden Ranking Open Edition, which evaluates the scientific performance of research institutions, on OpenAlex data. This represented a departure from the traditional Leiden Ranking, which is based on closed data from the Web of Science (Van Eck *et al.*, 2024 ▸). Furthermore, the French government has endorsed OpenAlex as an alternative to proprietary platforms (Ministry of Higher Education, Research and Innovation, 2021 ▸; Ministry of Higher Education, Research and Innovation, 2024 ▸). Finally, OpenAlex’s recent £2.9 million award from the Wellcome Trust to index research grants from funders globally (Wellcome, 2025 ▸) provides a compelling incentive for adoption by major facilities. This is particularly true for Diamond Light Source, which is also supported by Wellcome Trust funding and benefits from aligning with a key funder’s data infrastructure initiative.

### 2.2. Overview of OpenAlex

OpenAlex is an open catalogue of the global research system (Priem *et al.*, 2022 ▸). It encompasses scholarly entities (each with a unique OpenAlex ID) that are connected to one another in a heterogeneous directed graph. These entities are:

(i) Works: more than 240 million scholarly outputs such as journal articles and datasets.

(ii) Authors: around 90 million individuals responsible for producing Works.

(iii) Sources: approximately 249000 platforms (*e.g.* journals, repositories) that host Works.

(iv) Institutions: about 109000 organizations with which Authors are affiliated.

(v) Topics: the 4516 research areas of Works. Refer to Section 2.3 for a more detailed description.

(vi) Publishers: around 10000 organizations that disseminate Works.

(vii) Funders: around 32000 organizations that finance research.

OpenAlex is continuously updating its repository to provide a comprehensive index of scholarly outputs. The above figures for the various scholarly entities are accurate as of October 2025.

In order to tag Works with Topics in real time, the OpenAlex team developed a machine-learning model (Section 2.4) to perform automatic topic classification of the Works. This model is the main focus of this paper.

### 2.3. Topic classification scheme

**Figure 1:**
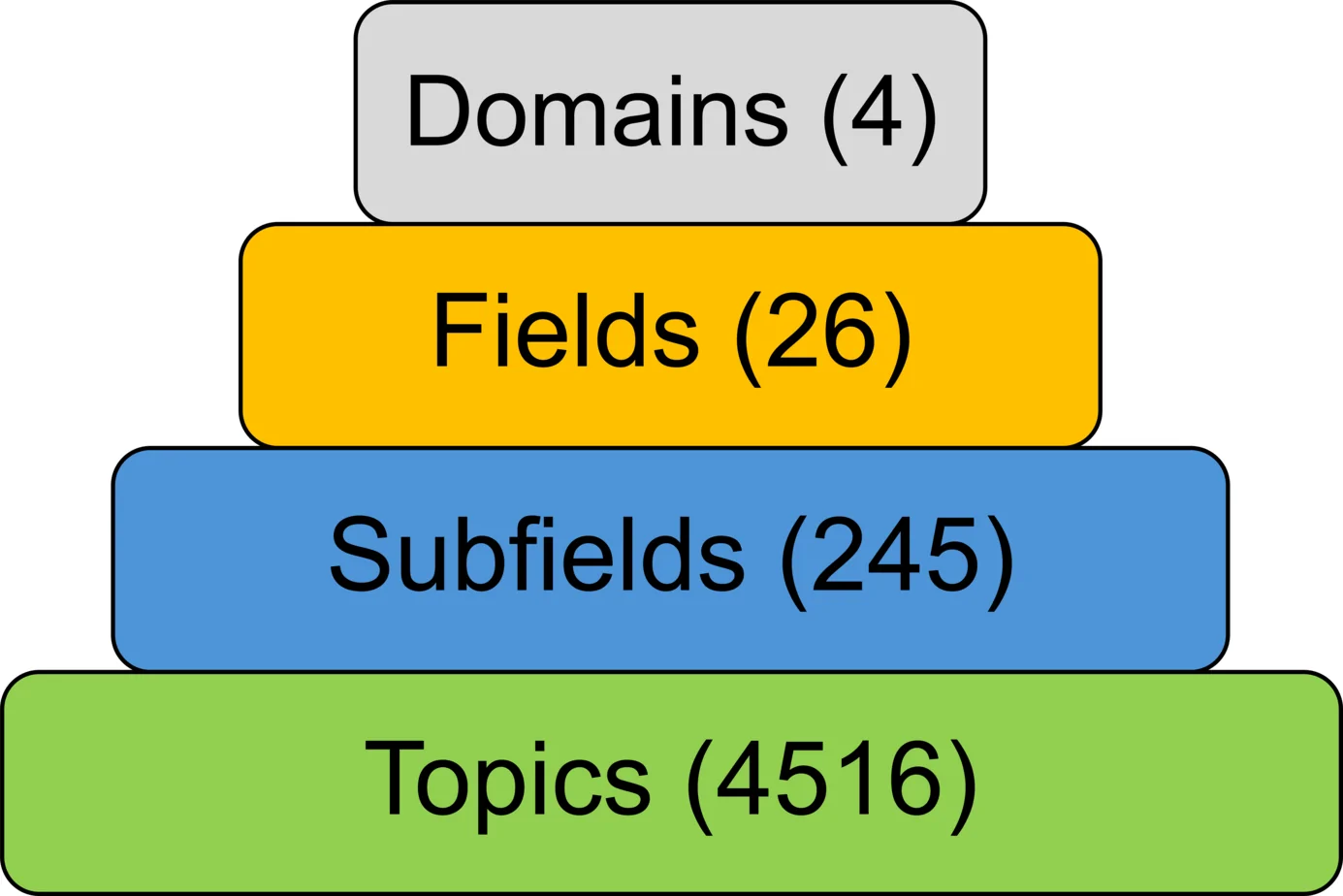
The classification follows a four-level hierarchy, from the most granular level of Topics to Subfields, and then to Fields, and finally to Domains.

The Topics are the most granular tier within a hierarchical classification system and are subordinate to Subfields, which are grouped into Fields, which are categorized by the broadest level of Domains. This hierarchy is illustrated in Fig. 1 ▸. As of October 2025, there are 4516 Topics, 245 Subfields, 26 Fields, and 4 Domains. An example of a Topic along with its hierarchy and metadata is shown in Table 1 ▸.

The classification was developed by the OpenAlex team and the Centre for Science and Technology Studies (CWTS) at Leiden University in The Netherlands.

The CWTS team first applied the Leiden algorithm (Traag *et al.*, 2019 ▸) in conjunction with what they refer to as the extended direct citation clustering approach (Waltman *et al.*, 2020 ▸) to a snapshot of the OpenAlex repository, clustering the 71 million scholarly outputs based on the 1715 million citation links between them (Van Eck & Waltman, 2024 ▸). The idea is that publications strongly connected by citation links are likely to be within the same research area. This resulted in a three-level hierarchical classification, from ‘micro level’ at the most granular level, to ‘meso level’, and finally to ‘macro level’ at the highest level. Since clustering is an unsupervised technique, even after the clusters had been identified, it was not clear which cluster corresponded to which research area. So, for each ‘micro level’ research area, the OpenAI GPT-3.5 Turbo (https://platform.openai.com/docs/models/gpt-3-5-turbo) model was provided with the titles of the 250 most cited publications in the area and asked to return a short label (of at most three words), a long label (of at most eight words), a list of keywords, a summary, and a Wikipedia (https://www.wikipedia.org) Uniform Resource Locator (URL). This approach was also applied to the ‘meso level’ and ‘macro level’ research areas, but the results were not deemed satisfactory by the CWTS team.

The resulting set of classifications forms the basis for OpenAlex’s topic classification. Specifically, the labelled ‘micro level’ classifications are now what OpenAlex is referring to as Topics. Using a Large Language Model (LLM), each Topic was then assigned to the Subfields, Fields, and Domains, which are based on Scopus’s All Science Journal Classifications (ASJC) (https://service.elsevier.com/app/answers/detail/a_id/15181/supporthub/scopus/) categories, culminating in the classification shown in Fig. 1 ▸. The OpenAlex team is committed to updating and improving this classification scheme. However, the Topics are expected to remain stable in the near term.

The complete set of classifications for all 71 million scholarly outputs was published on Zenodo (Van Eck, 2024 ▸), and forms the training dataset for the OpenAlex topic classification model, which is discussed in the next section.

### 2.4. Topic classification model

The OpenAlex team built a machine-learning model (refer to Appendix *A*[App appa] for more details on the model architecture) to predict Topics for incoming publications in real time. The model takes in the title, abstract, outgoing citations, and journal name to produce a vector 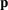, where each element *p*_*i*_ is the independent probability that Topic *i* is relevant to the input Work. The model selects the *TopK* Topics associated with the highest probabilities in 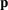. *TopK* is an adjustable hyperparameter; in our specific application, *TopK* is set to 3 because that is the default value selected by OpenAlex. Finally, a series of confidence-based post-processing steps are applied to the *TopK* predictions. These heuristics evaluate the relative probabilities of the selected Topics to filter out lower-confidence results, ensuring a final output of high relevance. The model was trained on the classifications performed by the CWTS team. The model artefacts and training data can be found on Zenodo (OpenAlex, 2024*b* ▸).

On 4 November 2025, OpenAlex transitioned to a new codebase, nicknamed ‘Walden’ (Priem, 2025 ▸). This update included an improved topic classification algorithm. The changes were not large and did not involve retraining of the underlying neural network, as confirmed by the OpenAlex team. Our work utilizes the pre-Walden version of the topic classification model. Given the nature of the update, the older model’s outputs remain sufficient for the purposes of this study.

## 3. Methodology

We repurposed the OpenAlex topic classification model to classify the experiment proposals. This is feasible because the proposals share structural elements with journal articles, such as titles, abstracts, and references. However, the proposals’ lack of an associated journal name is a key difference. To address this, the Scientific Discipline was substituted based on the rationale that journal names frequently include the relevant domain anyway. Furthermore, since the model transforms journal names into embeddings (explained in Appendix *A*[App appa]), using Scientific Disciplines as a substitute will still produce embeddings with the essential semantic meaning. An analysis of the effect of this substitution can be found in Section 4.4, which reveals that this substitution is justified.

Our methodology is also supported by recent work (Fenner, 2025 ▸) showing that the OpenAlex model can be successfully repurposed for different types of content.

The first step was to collect the necessary data to serve as input for the model: titles, abstracts, references, and Scientific Disciplines (Section 3.1). Supplementary metadata was also collected as a way to further augment the model’s performance by trying different combinations of inputs (Section 3.2). Finally, an evaluation exercise was carried out to assess the model’s performance (Section 3.3).

### 3.1. Data collection

As mentioned earlier in Section 1.2, the proposal documents themselves are not publicly available. However, the experiment reports are largely accessible from the ESRF data ecosystem (refer to Appendix *B*[App appb] for more details), and the titles and abstracts of the reports are very often identical to those of the proposals. The content and list of references within the reports, which are extracted in a structured format using GROBID (GROBID, 2008–2025 ▸), are also expected to be very similar. For context, GROBID is a machine-learning tool that parses and converts PDF documents into structured XML/TEI files. We chose it because it seems well suited for this type of task (Meuschke *et al.*, 2023 ▸; Siebenschuh *et al.*, 2025 ▸) and also offers an optional integration with Crossref to correct and complement the extracted data, enabling us to retrieve DOIs for references where they were initially missing. Since each proposal could be associated with multiple experiment reports, we joined all unique titles into a single text block, did the same with the abstracts, and compiled the references into a single list.

**Figure 2:**
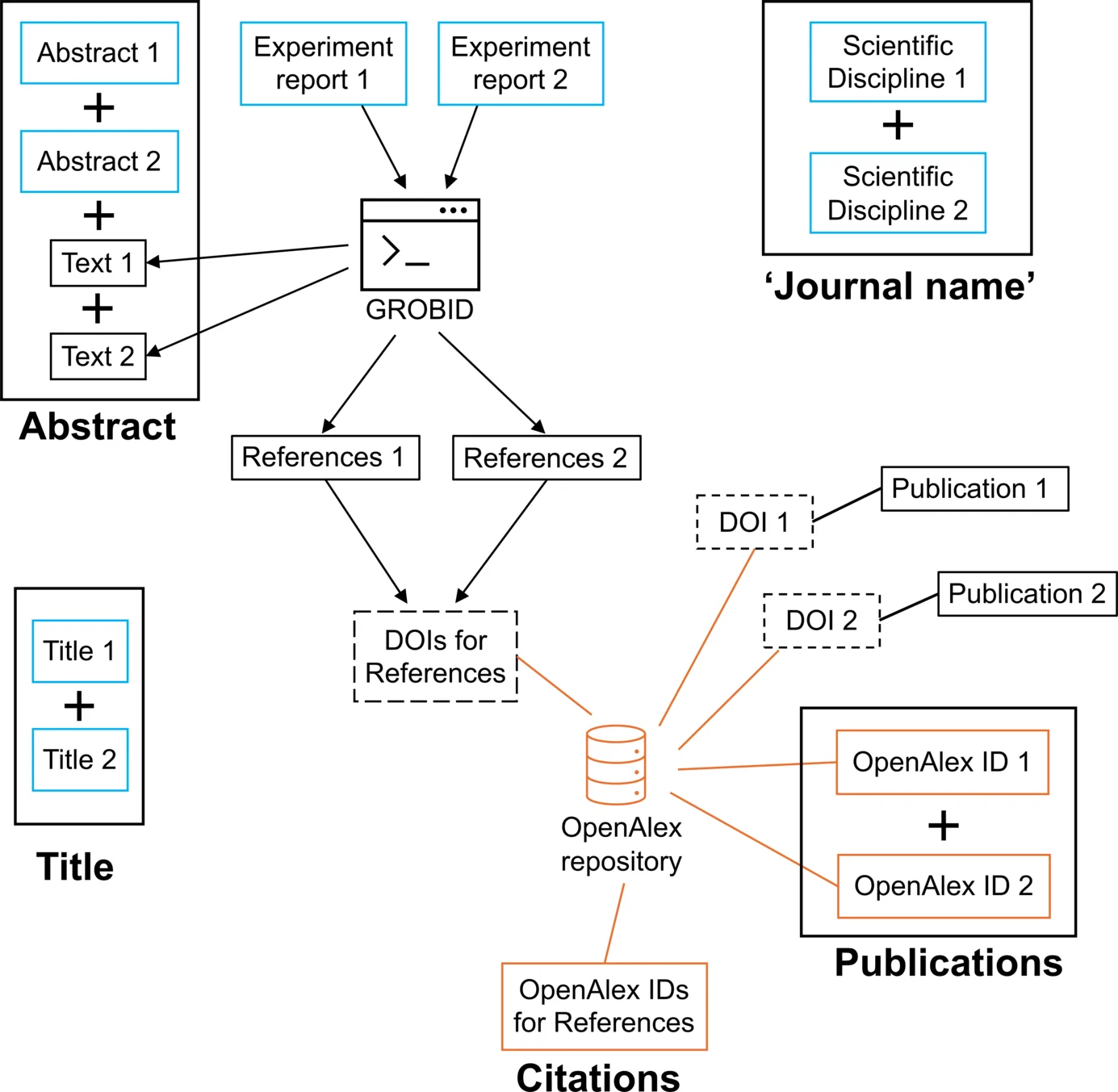
This figure illustrates the processing of the raw metadata pulled from the ESRF data ecosystem. The Titles are joined to form a singular Title. GROBID was used to process the experiment reports into structured texts and citation information. These structured texts are combined with the Abstracts to form the Abstract. The same was done with the Scientific Disciplines, which were used as a proxy for ‘Journal name’. The references extracted using GROBID include the DOIs, allowing them to be linked to their OpenAlex IDs via the OpenAlex repository. This gave the Citations. Similarly, the publications were mapped to their OpenAlex IDs via DOIs, resulting in the collective Publications. The dashed squares represent unique identifiers that link the various elements. The Title, Abstract, Citations, ‘Journal name’, and/or Publications were the inputs to the OpenAlex model for topic classification of the proposals.

A total of 5384 proposals were collected, with the following associated metadata (refer to Fig. 2 ▸):

(i) Title: textual information accessed from the ESRF data ecosystem.

(ii) Abstract: textual information accessed from the ESRF data ecosystem, together with the content extracted from the experiment reports using GROBID.

(iii) Citations: publications cited as reference in the experiment report; their DOIs were extracted using GROBID and linked to their counterpart OpenAlex IDs via the OpenAlex repository.

(iv) ‘Journal name’: the Scientific Disciplines accessed through the metadata tags assigned to the experiment sessions in the ESRF data ecosystem.

(v) Publications: the DOIs of publications were traced from their origin proposals using the unique proposal numbers, and then linked to their corresponding OpenAlex IDs via OpenAlex.

### 3.2. Applying the model

A total of two different combinations of data were passed into the model, with each reflecting the data available at different stages of the science lifecycle:

**Combination 1:** the four core inputs as defined in Section 3.1 (**Title**, **Abstract**, **Citations**, **‘Journal name’**). This is what can be expected to be available at the proposal submission stage.

**Combination 2:** the four core inputs plus the **Publications** associated with the proposals. All of this information would be available at the last stage of the science lifecycle, when the experiments have concluded and the results have been written up and published.

The first combination reflects the data available to the planned live service, while the second could be used for back-classification of historical proposal documents that have been archived. Both applications of the model were carried out for all 5384 proposals, resulting in two sets of data that have been uploaded to the GitHub repository (Tan, 2024–2025 ▸). Since investigating the feasibility of the live service was the immediate priority, the evaluation exercise was conducted for only the first combination.

### 3.3. Evaluation exercise

The evaluation exercise had two objectives:

(i) Primary objective: **evaluate the feasibility of a live service to perform topic classification in real time**. The idea of the live service is to provide the beam-time applicants with a list of possible Topics for selection. Given that the suggestions will be verified on the spot by the applicants, the service is considered successful if at least one Topic is correct. If not, the live service must still accurately narrow down the available choices by providing a sufficient set of accurate Subfields and Fields, enabling the applicants to select the correct Topics themselves.

(ii) Secondary objective: **evaluate the feasibility of performing topic classification on previously submitted proposals**. The requirements for this task are much stricter because there is no easy way to verify the predictions, unlike in the case of the live service. Consequently, both the model’s Precision (the fraction of Topic predictions that are actually relevant) and Recall (the fraction of all relevant Topics that were successfully captured) must be very high, in spite of the absence of publication information due to the choice of Combination 1 (see Section 3.2), to prevent frequent misclassifications from being introduced into the historical data.

Although the model was applied to all 5384 proposals, only 209 of them had the full set of input data. Specifically, the proposal must fulfil all the following criteria:

(i) Have a Title.

(ii) Have an Abstract.

(iii) Have at least one reference with a DOI, *i.e.* at least one Citation.

(iv) Have an assigned Scientific Discipline, which is used as a proxy for ‘Journal name’.

The missing data are likely due to extraction errors by the GROBID tool and gaps or omissions within the ESRF data ecosystem, and there is no reason to believe that the missing data are systematically different from the available data. In a live service scenario, the Title, Abstract, and Scientific Discipline would be available (as they are usually mandated by facilities), so these 209 proposals are fairly representative of what can be expected. The only caveat is that Citations are not guaranteed to be available (see discussions in Sections 4.4 and 5).

**Figure 3:**
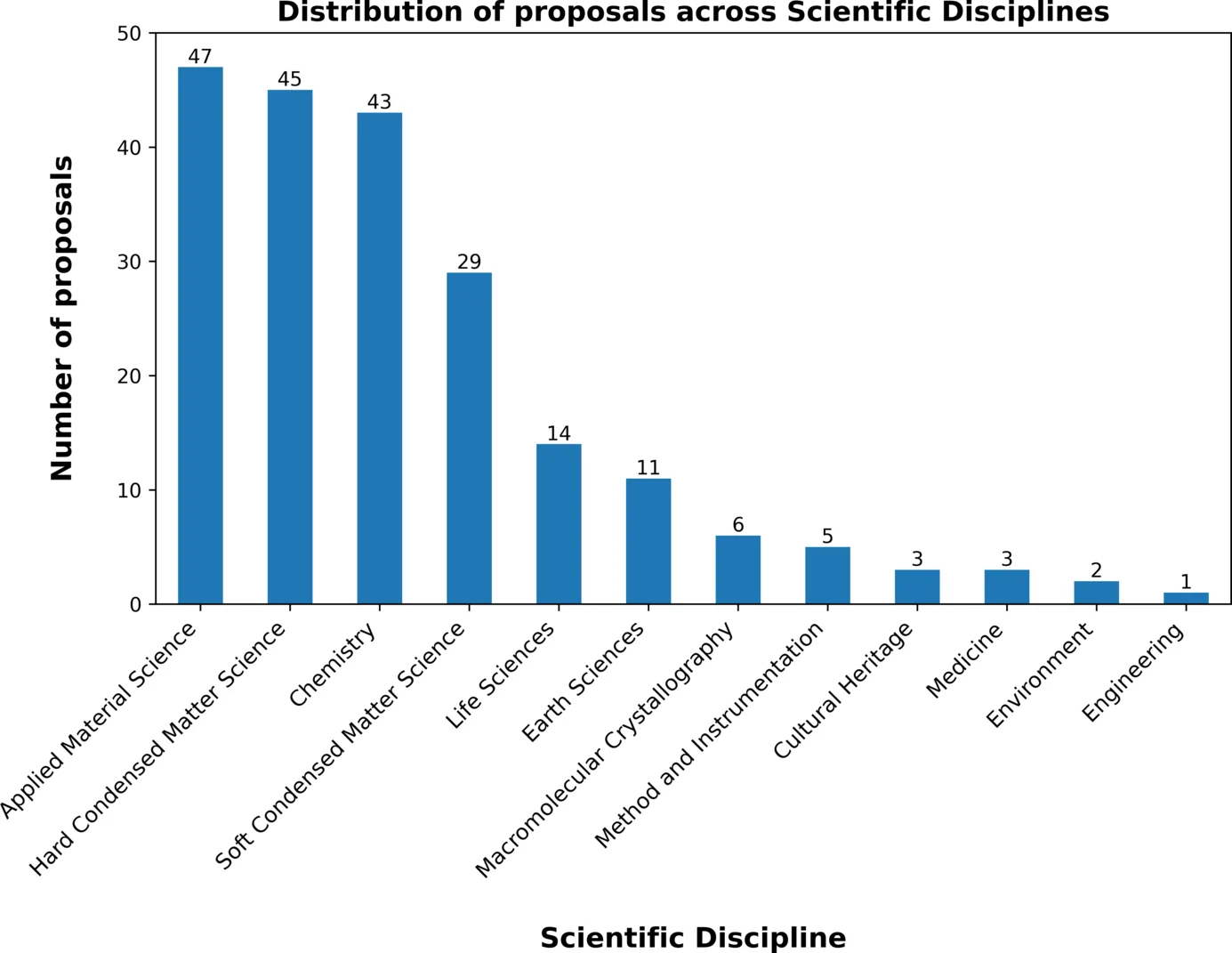
Bar chart of the distribution of the 209 proposals used for the evaluation exercise across ESRF Scientific Disciplines.

**Figure 4:**
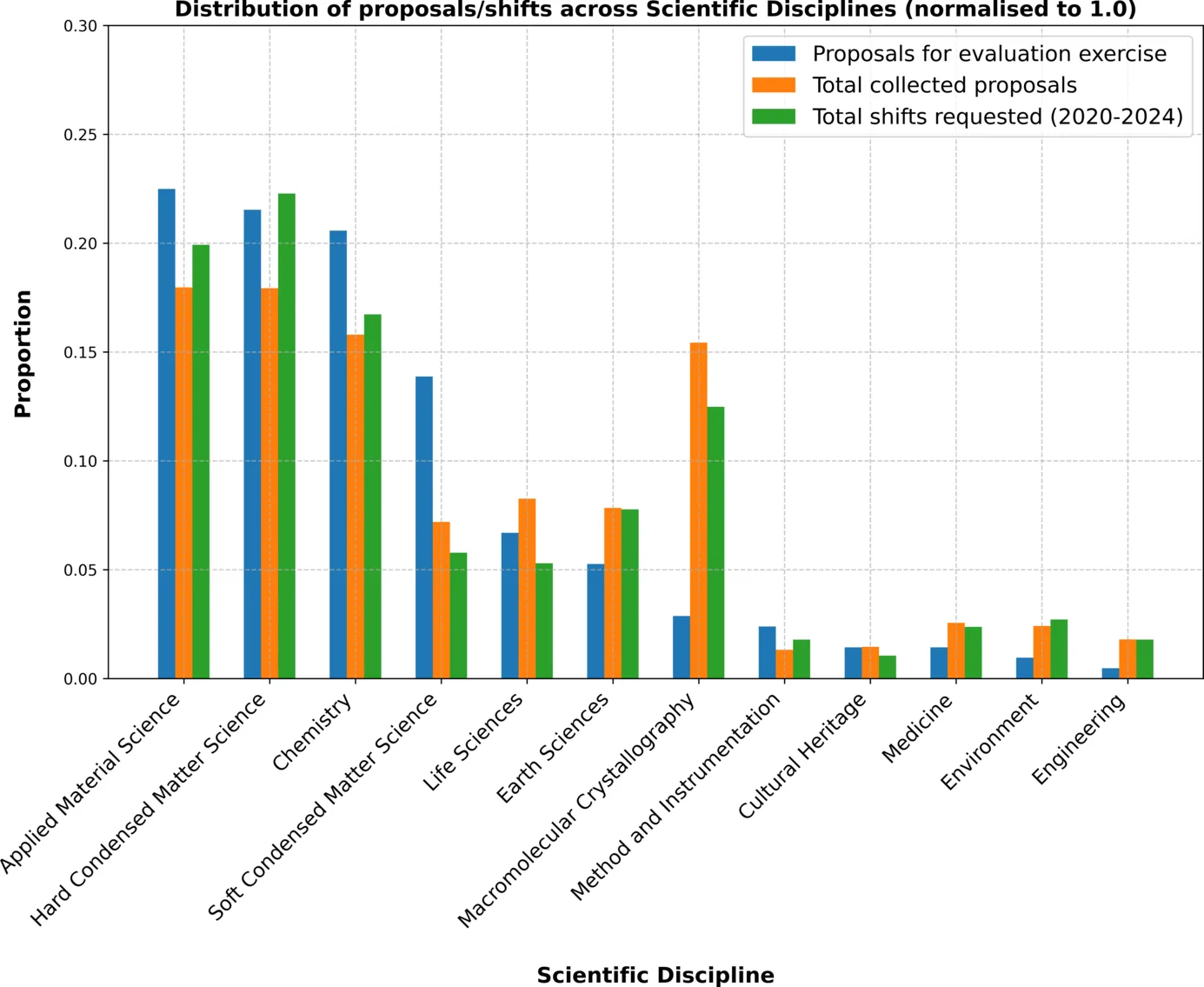
Normalized distributions of proposals and shifts requested across the Scientific Disciplines. The distributions for the 209 proposals being used for the evaluation exercise (which only covers the period from 2020 to 2024), the 5384 total collected proposals, and the total shifts of beam time requested from 2020 to 2024 are normalized and compared in this plot. The comparison shows that the distributions are largely similar, with ‘Macromolecular Crystallography’ proposals possibly being under-represented within the proposals used for the evaluation exercise.

Furthermore, the distribution of these proposals across the Scientific Disciplines (Fig. 3 ▸) roughly matches that of the original 5384 collected proposals (Fig. 4 ▸). Since the actual distribution of all submitted proposals is not publicly available, the total number of requested beam-time shifts from 2020 to 2024 was used as a proxy for the true distribution. This beam-time shift data, calculated from the 2020–2024 ESRF Highlights documents (ESRF, 2020 ▸; ESRF, 2021 ▸; ESRF, 2022 ▸; ESRF, 2023 ▸; ESRF, 2024 ▸), also yield a close match to the reviewed proposals. This suggests that the evaluation set is a representative sample, with two key caveats:

(i) The ‘Macromolecular Crystallography’ proposals appear to be under-represented. This is likely because many of them are Block Allocation Group (BAG) proposals. Since these BAG proposals bundle many experiments into a single proposal, they represent a high volume of beam time despite the low proposal count. Separately, it is worth noting that their wide-ranging scope would make them harder to classify than more specialized non-BAG proposals.

(ii) The sample size is small for all Scientific Disciplines except ‘Applied Material Science’, ‘Hard Condensed Matter Science’, ‘Chemistry’, and ‘Soft Condensed Matter Science’.

For any statistical analysis grouped by the Disciplines, it would be better to consolidate the proposals with small sample sizes into an ‘Others’ category (Section 4).

Finally, reviewing 209 proposals is just a much more manageable task that still provides enough information to achieve the objectives outlined above.

Eighteen domain experts (all of whom are authors of this paper) were selected as reviewers and assigned to the 209 selected proposals based on the Scientific Disciplines. To mitigate individual subjectivity and ensure scientific rigour, each proposal was independently evaluated by three reviewers. This also enabled the analysis of the inter-reviewer agreement; a high level of agreement between the reviewers, whether concluding the predicted Topics are relevant or irrelevant, would serve as a strong validation signal. Conversely, low agreement or high uncertainty would suggest that the title and abstract were poorly written, meaning that the model cannot be blamed for misclassification.

The full list of reviewers and their assigned Scientific Disciplines (ESRF categorization, see Section 1.1) can be found in Table 2 ▸.

For each proposal, the reviewer was provided with the title, abstract, references, Scientific Disciplines, the URLs of the experiment reports, and the predicted Topics, Subfields, and Fields. They were instructed to read the title and abstract (and the supplementary information if needed), and assess the Topics by selecting ‘Relevant’, ‘Not relevant’, or ‘Unclear’ from a drop-down menu.

If all Topics were rejected as ‘Not Relevant’ and/or ‘Unclear’, they would move on to evaluate the Subfields, which are one level higher in the classification scheme. If all Subfields are deemed to be ‘Not relevant’ and/or ‘Unclear’ as well, they proceed to review the Fields, which are another level up in the hierarchy. This tiered evaluation ensured the accuracy of the broader categories, allowing users to more easily narrow down and select the appropriate topics, even if the initial predicted list was rejected. Crucially, this approach made the evaluation process lightweight and minimized the substantial workload inherent in having three reviewers evaluate each proposal, albeit at the cost of forfeiting the complete evaluation of all underlying Subfields and Fields.

**Figure 5:**
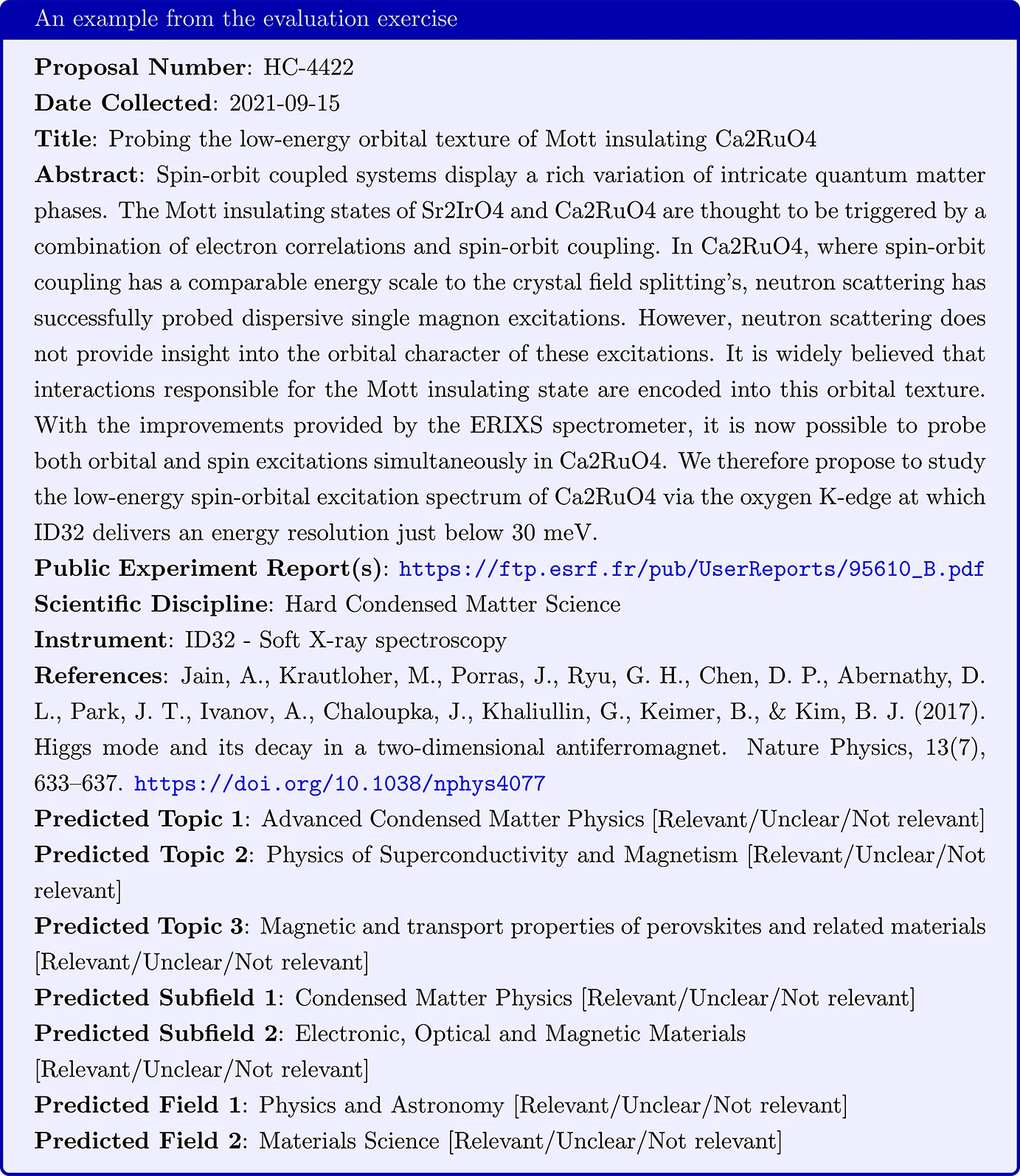
An example from the evaluation exercise.

To illustrate the review interface, a ‘Hard Condensed Matter Science’ proposal (Chang *et al.*, 2024 ▸) is presented as a representative example of the metadata and model outputs evaluated by the reviewers – see Fig. 5 ▸. In this particular case, the reviewer had evaluated all Topics as ‘Relevant’. Hence, there was no need to evaluate the Subfields and Fields.

Note that this evaluation exercise does not capture any information about Topics that are relevant but not predicted by the model at all (false negatives). To reduce reviewer workload, we prioritized evaluating Precision over Recall. This trade-off aligns with our primary objective to investigate feasibility of a live service, in which real-time validation by domain experts is available and the *TopK* hyperparameter (see Section 2.4) can be increased to boost Recall. In addition, the variance in reviewer responses would likely be quite high if they were to identify missing Topics. Some reviewers may be more lenient and only identify the most glaring omissions, while others may be more meticulous and identify a much larger number of missing Topics. This would make the Recall value less reliable and less actionable than that of Precision, which is more straightforward for reviewers to evaluate and for us to interpret. As a result, we are relying on the proposal-based Precision value to gauge model performance against our secondary objective for now, with the expectation that the Recall value will be investigated in future work when the live service allows proposal authors to validate predictions in real time.

## 4. Evaluation results and analysis

The bulk of the evaluation datapoints is derived from the Topic predictions. While most proposals have three Topic predictions, Proposal EV-514 (Ciani *et al.*, 2026 ▸) is the sole exception, with only two. This discrepancy occurs because the model’s post-processing stage (see Section 2.4) can filter out candidates from the initial top three. This leads to a total of 626 Topic predictions across the 209 proposals. Given that each proposal is assigned three reviewers, the set of Topic ratings comprises 1878 datapoints. A minority of proposals required deeper review when their Topic predictions were uniformly ‘Unclear’ and/or ‘Not relevant’ (see Section 4.2.1 for details). This necessitated an evaluation of the Subfields and, in some cases, the broader Fields, yielding an additional set of datapoints. This brings the total number of datapoints resulting from the evaluation exercise to 2110.

We begin by examining the inter-rater agreement among the independent reviewers (Section 4.1). We then analyse the performance of the model primarily at the Topic level (Section 4.2). However, we also extend this analysis to the Subfields and Fields in those specific cases where the Topics predictions completely fail to capture the essence of the proposal (Section 4.2.1). In addition, we compare our reviewer evaluations with the model’s own confidence score in Section 4.2.2. We also summarize and discuss the observations and comments made by the reviewers throughout the evaluation exercise (Section 4.3). Lastly, we carry out an ablation study in Section 4.4.

The ratings are converted to a numerical value to facilitate the calculation of the inter-rater agreement and model performance metrics: Relevant – 1; Unclear – 0.5; Not relevant – 0.

Statistical measures are also calculated for each Scientific Discipline. As noted in Section 3.3, the Disciplines with limited sample sizes are aggregated into an ‘Others’ category. These Disciplines are: ‘Earth Science’, ‘Environment’, ‘Cultural Heritage’, ‘Life Sciences’, ‘Medicine’, ‘Engineering’, ‘Methods and Instrumentation’, and ‘Macromolecular Crystallography’.

### 4.1. Inter-rater agreement

The inter-rater agreement quantifies the degree of consistency in the reviewers’ assessments. A high agreement score suggests that the proposals (or more specifically, the titles and abstracts) are written with sufficient clarity for domain experts and that the Topics are well defined and consistently interpreted across the reviewers. In this analysis, we chose to use three distinct metrics to provide a robust and comprehensive measure of agreement: Fleiss’ kappa, Randolph’s kappa, and Krippendorff’s alpha.

Fleiss’ kappa (Fleiss, 1971 ▸) is an extension of Cohen’s kappa (Cohen, 1960 ▸), which can only be used for two raters (McHugh, 2012 ▸), that measures the extent to which the agreement among the multiple raters exceeds what would be expected by random chance,
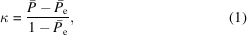
where 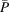 is the mean proportion of all ratings that are in agreement for each sample across all raters, and 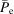 is the mean proportion of agreement that would be expected to occur purely by chance, calculated using the observed distribution of ratings. The numerator 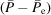 would then represent the degree of agreement observed above what is expected by chance, while the denominator 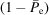 is the maximum possible amount of agreement above chance.

Randolph’s kappa (Randolph, 2005 ▸) is a modification of Fleiss’ kappa that assumes a uniform distribution of rating categories as the benchmark 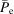, rather than the marginal sample distribution. Both Fleiss’ kappa and Randolph’s kappa are calculated for nominal categorical data. As a result, they make no distinction between the severity of different types of disagreements, treating the mathematical distance between any two distinct categories, such as ‘Relevant’ versus ‘Unclear’ and ‘Relevant’ versus ‘Not relevant’, as identical.

In contrast, Krippendorff’s alpha (Krippendorff, 2019 ▸) is capable of assessing data measured on any scale, including nominal. When applied to ordinal data, it incorporates a weighted measure of disagreement, which correctly reflects the magnitude of disagreements between raters.

Fleiss’ kappa and Randolph’s kappa were calculated using the statsmodels library (Seabold & Perktold, 2010 ▸), while Krippendorff’s alpha was calculated using the Fast Krippendorff Python package (Castro, 2017 ▸). The results are summarized in Table 3 ▸.

All three metrics range from −1 (systematic disagreement) to +1 (perfect agreement), with 0 representing chance agreement, *i.e.* the reviewers made their ratings as if completely at random.

The consistently positive but relatively low values calculated in Table 3 ▸ indicate a general consensus among the reviewers, albeit with a notable degree of variance. This highlights the inherent subjectivity and difficulty of classifying scientific text even for domain experts, as shown by the consistency of these scores across all Disciplines.

### 4.2. Model performance

We now proceed to use the reviewer evaluations as a proxy for the models’ performance. Specifically, we take the average of the three reviewers’ scores for each prediction and interpret the resulting values primarily through the lens of our two objectives defined in Section 3.3. Note that, due to the three-point evaluation scale, possible mean scores are restricted to the set
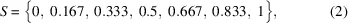
where each value *s* ∈ *S* represents a possible mean reviewer score. We establish *s* > 0.5 (which is equivalent to *s* ≥ 0.667) as the baseline threshold for ‘correctness’. We then evaluate the model’s performance against increasingly stringent benchmarks as *s* moves toward 1, allowing us to observe how performance fluctuates under stricter consensus requirements.

Our primary objective is to assess the viability of a hypothetical live service. Recall that we consider the application successful if it generates at least one relevant Topic. Our secondary objective is to investigate the feasibility of retrospective topic classification on past proposals using the model.

Referring to Table 4 ▸, the proportion of proposals with at least one ‘correct’ Topic prediction is high across all Scientific Disciplines. Even the proportions for ≥2 Topics are consistently high enough across the Disciplines for the model to be useful in a live service setting. However, the proportion of proposals with three ‘correct’ Topics is low. These observations hold for the different consensus requirements, with performance unsurprisingly degrading as τ (the threshold for ‘correctness’) increases. This means the model rarely achieves perfect Precision; not all Topics it identifies as relevant are truly so. To quantify this, we calculate the proposal-based Precision using the following formula,
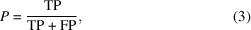
where TP is the number of true positives (predicted Topics with *s* ≥ τ = 0.667, which is the baseline threshold for ‘correctness’) and FP is the number of false positives (predicted Topics with *s* < τ = 0.667). By calculating this ratio for every individual proposal and averaging the results, we arrive at an overall Precision score of 56.0%.

This relatively low Precision is manageable in a live setting where proposal authors can verify predictions in real time, but it becomes unacceptable for autonomous applications lacking human oversight. In addition, this evaluation exercise fails to account for Recall, which measures the model’s ability to identify all truly relevant Topics. This lack of data regarding false negative rates further weakens the case for autonomous use.

As noted above, the post-processing stage of the model (see Section 2.4) introduces the possibility of proposals with fewer than three total Topic predictions. Proposal EV-514 is the only such case among the 209 proposals, with two predicted Topics. All three reviewers deemed these two Topics as ‘Not relevant’, with one reviewer even pointing out that the extracted references do not match the proposal [see point (iv) in Section 4.3]. Hence, this outlier proposal is automatically accounted for in Table 4 ▸.

Analysis of the model’s classification failures reveals three primary edge cases:

(i) Niche experimental scope: proposals detailing unique experiments that likely lack the common linguistic markers of the correct classifications.

(ii) Broad categorical range: BAG proposals that span a wide variety of themes, making them difficult to pin to specific Topics. This was anticipated in Section 3.3.

(iii) GROBID extraction artefacts: technical failures where the GROBID tool incorrectly extracted non-existent references from the source documents, introducing noise into the classification process. This was noted by several reviewers (Section 4.3).

#### 4.2.1. Subfield and field recovery rates

In cases where all Topics are deemed ‘Unclear’/‘Not relevant’ by a reviewer, they were instructed to evaluate the Subfields. If the same happens for the Subfields, the reviewer proceeded with evaluating the Fields.

Of the 73 instances where no Topics were found ‘Relevant’, 55 (75.3%) contained at least one relevant Subfield. Among the remaining 18 cases, 8 included at least one ‘Relevant’ Field. This suggests that even when granular Topic predictions fail entirely, the broader taxonomic hierarchy (Subfields and Fields) serves as a reliable fallback; in a live service context, this allows the model to guide users towards the correct area in 86.3% of these initial failure cases.

#### 4.2.2. Root mean square error

We can also compare the mean reviewer scores with the model’s own confidence scores. Recall that each predicted Topic *i* has an associated probability of relevancy *p*_*i*_, which the model uses to select the top three predictions for each proposal. Using these two sets of scores, we calculate the root mean square error to be 0.565. This large error is unsurprising, given that the top predictions often yield *p*_*i*_ > 0.9, which is functionally equivalent to all three reviewers marking the Topic as ‘Relevant’. In contrast, the inter-rater agreement scores and the values calculated in Table 4 ▸ indicate that such consensus is actually quite uncommon in practice.

### 4.3. Reviewer comments

The reviewers were given the option to leave comments for each proposal during the evaluation exercise. There were several common and important observations that we have summarized below:

(i) The keywords and descriptions assigned to each Topic are sometimes inconsistent with the Topic name, creating confusion about the Topic’s actual meaning. This concern was raised during the initial stage of the evaluation exercise and resulted in the prompt removal of the descriptions and keywords to mitigate the issue.

(ii) The Topic names themselves are often vague. For example, Topic 10413, ‘High-pressure geophysics and materials’, seems to be a combination of two distinct areas: materials science at high pressure and high-pressure geophysics. Unfortunately, the keywords and descriptions could not be used to clarify meaning of the Topic name due to the bullet point above.

(iii) The model occasionally prioritized peripheral terminologies over the core subject matter of a proposal, resulting in incorrect classifications. For example, in Proposal HC-5077 (Dewaele *et al.*, 2025 ▸), the mention of a ‘laser-heated diamond anvil cell’ was interpreted as a direct focus on diamond materials research, despite the fact that the word ‘diamond’ is merely a descriptive term for a type of scientific device. This indicates that the model failed to differentiate between a research subject and an instrument descriptor.

(iv) GROBID made unexpected mistakes with some of the experiment reports. For example, GROBID extracted three non-existent references from Proposal ES-1032 (Brugger, 2025 ▸). Fortunately, the model was still able to make good predictions in that particular case. In addition, only 5 (2.39%) of the 209 proposals were singled out by the reviewers as having seemingly incorrect references. This suggests that GROBID parsing error is not common for bibliographic data.

### 4.4. Ablation study

In this section, we examine the individual contributions of each feature (Title, Abstract, Citations, and Scientific Discipline) to the model output. In the absence of ground truth labels for the proposals, we rely on the output variance rate as a proxy for feature importance. Specifically, we quantify the independent influence of each feature by calculating the percentage of proposals that yield divergent outputs when the feature is excluded (see Table 5 ▸).

The results suggest that the Abstract has the most influence on the model output. This contrasts with the ablation study carried out by OpenAlex (OpenAlex, 2024*a* ▸), which indicated that the Citation feature is the most important factor in model performance. The reason for this discrepancy is not clear, but it could be due to the different metric being used for feature importance, as the OpenAlex team had access to ground truth labels. Nevertheless, both studies are in agreement that the Title, Abstract, and Citations features have noticeable influence on model performance. Fortunately, we can expect experiment proposals to always have the Title and Abstract as synchrotron facilities usually mandate their inclusion. In contrast, proposals do not necessarily contain Citations, and a total absence will significantly degrade model performance. Interestingly, the correlation between the number of references and the mean score is very weak (Pearson = −0.0136, Spearman = 0.0271), which suggests that, while a complete lack of Citations is detrimental, sparse referencing is still acceptable.

Our findings also indicate that the Scientific Discipline had no influence on the final model output in most cases. In the minority of cases in which it led to a different Topic prediction, the Topic was generally deemed relevant by the domain experts (see Table 6 ▸), suggesting that the Scientific Discipline only plays a small role in steering the model in the right direction. This mirrors the findings of the OpenAlex team, which reported that the journal name feature provides a weak signal to the model (OpenAlex, 2024*a* ▸). Hence, while the Scientific Discipline provides only a marginal gain, our initial decision to use it as a functional proxy for journal names is empirically justified.

## 5. Summary

This paper investigates the feasibility of applying the OpenAlex model and its classification scheme to synchrotron experiment proposals. We chose ESRF proposals as the case study for two main reasons: first, ESRF is actively interested in implementing a live topic classification service based on the OpenAlex ecosystem; and second, the openness of their data ecosystem allows for the necessary study and publication of results. Crucially, the inherent similarity of synchrotron experiment proposals means that our findings are generalizable across most, if not all, synchrotron facilities.

We applied the OpenAlex model to a total of 5384 ESRF experiment proposals, and carried out an evaluation exercise with 18 domain experts on 209 of them. Each proposal was independently assessed by three reviewers. We then conducted an analysis of the results, mainly by considering the inter-rater agreement and the reviewers’ assessment of the model’s predictions. We found that:

(i) The model is accurate enough for a live service implementation, in which the predictions would be reviewed on the spot by the proposals’ authors, who can be considered the final authorities on the subject matter of their respective proposals.

(ii) The model is not accurate enough to classify proposals without human expert oversight. This means that it should not be used for previously submitted proposals, where the authors would not be present to validate the results. The model particularly struggles with broad proposals that cover a range of topics, such as BAG proposals.

(iii) GROBID is a very powerful tool for extracting structured information from the experiment reports, but it occasionally fails in its parsing, which directly compromises the model’s predictions. Hence, the unstructured free text nature of the reports presents a significant bottleneck to the model’s performance. Future studies should investigate the use of alternative PDF parsing tools (Siebenschuh *et al.*, 2025 ▸).

(iv) The OpenAlex classification scheme provides a comprehensive and robust controlled vocabulary and taxonomy for experiment proposals. However, some of the descriptions and keywords do not seem to match their corresponding Topic labels and would have to be updated. Given that OpenAlex is still in active development, this is not expected to be a long-term issue. In the meantime, the descriptions and keywords can be excluded from any live service implementations.

We also note the following caveats:

(i) The proportion of ‘Macromolecular Crystallography’ proposals (among the 209 reviewed proposals) is under-represented relative to its expected frequency in the overall proposal pool.

(ii) The sample size is small for ‘Earth Science’, ‘Environment’, ‘Cultural Heritage’, ‘Life Sciences’, ‘Medicine’, ‘Engineering’, ‘Methods and Instrumentation’, and ‘Macromolecular Crystallography’ proposals. Aggregating these eight Scientific Disciplines into a single ‘Others’ category may obscure field-specific variance and limit generalizability of the model. Hence, model predictions for proposals under these Scientific Disciplines would have to be scrutinized more carefully. However, such proposals are unlikely to be encountered frequently during real-world model deployment, since they are inherently under-represented in reality.

(iii) The evaluation exercise does not capture information regarding relevant Topics that the model completely failed to predict (*i.e.* missing relevant Topics). Therefore, the metrics used to judge model performance in Section 4.2 are based on Precision rather than Recall values.

(iv) The model depends heavily on Citations, but there is no guarantee that proposals will include them. In such cases, the broader classifications (Subfields) can be used to guide the selection of Topics. This guided discovery workflow, described in Section 5.1, acts as a fallback option when Citations are completely absent. Fortunately, the model is robust against sparse referencing.

(v) The 209 proposals used for the evaluation exercise were selected for data completeness rather than at random. This non-random selection may introduce biases related to unforeseen variables, which would limit the representativeness of the evaluated sample set.

With these points in mind, we can recommend the implementation of a live topic classification service that uses the OpenAlex model and classification scheme, with a couple of suggestions to maximize the model’s performance as part of a live service:

(1) **Increase the number of predictions shown by the model**. The current Topic prediction mechanism utilizes a default *TopK* parameter value of 3, which restricts the model’s initial output to the three highest-scoring predictions. The subsequent heuristic post-processing filter, based on prediction score criteria defined by the OpenAlex team, may further reduce this number. The live service will present the model output as a selectable list to users. Since the users essentially function as domain experts validating the predictions, the system should prioritize Recall (the likelihood of a relevant Topic being present) over strict Precision at the initial suggestion stage. This can be achieved by increasing the *TopK* value and relaxing the thresholds applied in the post-processing step. This will present users with a broader set of candidate Topics for selection.

(2) **Transition to a structured proposal data entry system**. Users currently submit their proposals in the form of PDF documents containing free text. This makes it challenging to extract the necessary data in a structured format for input to the model, especially since the GROBID tool is not infallible at parsing the documents. Replacing the current free text entry with specific, predefined sections within the user submission portal bypasses the need for GROBID or any other PDF parser tool.

### 5.1. Live service

This discussion explores a theoretical implementation of this service, where GROBID and the OpenAlex ecosystem are integrated into a generic User Portal, which acts as a proxy for standard proposal submission platforms.

**Figure 6:**
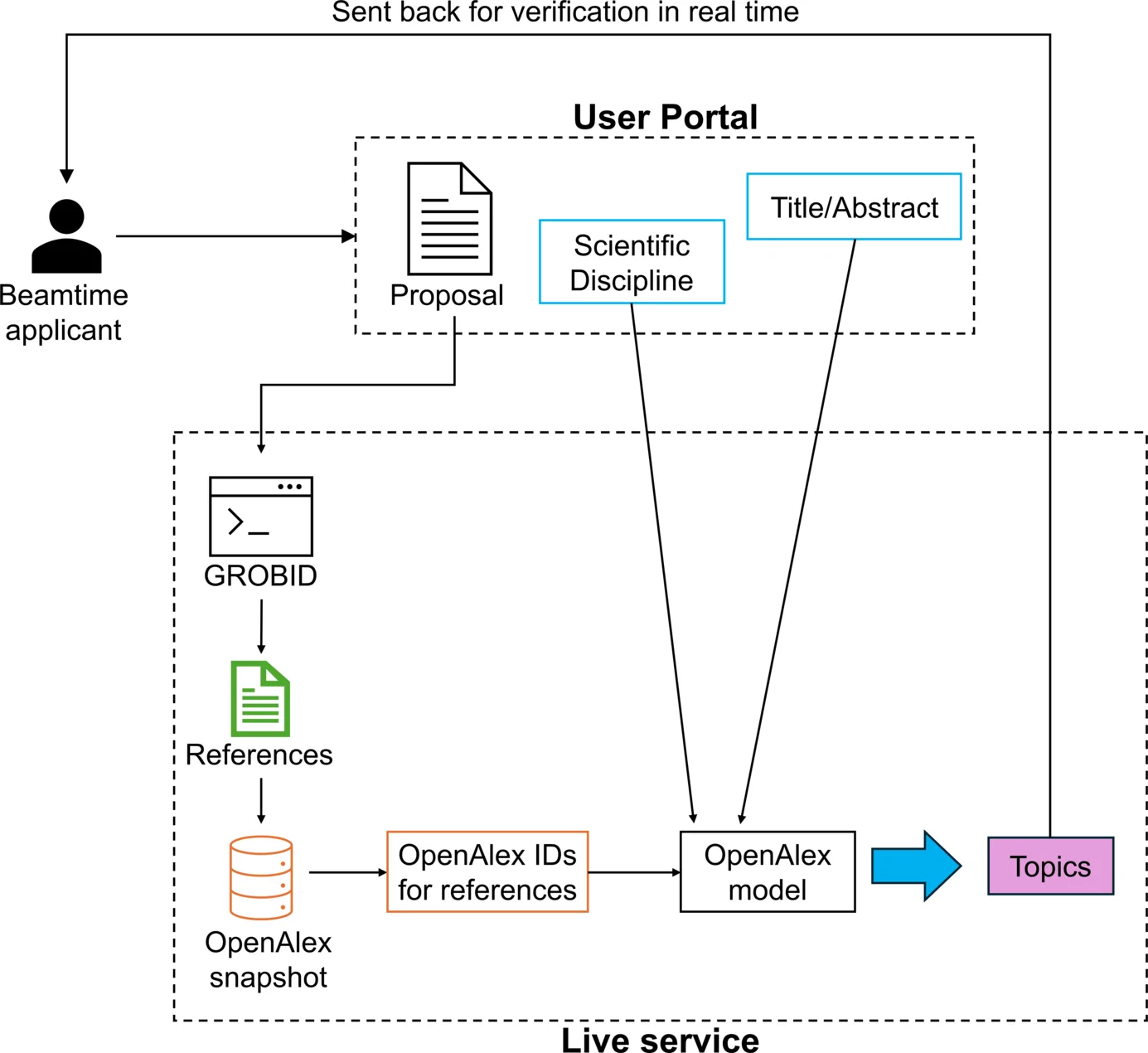
A visual representation of how a real-time topic classification service could be integrated into the beam-time application process. The DOIs of the references are extracted from the proposal document using GROBID, before being mapped to their corresponding OpenAlex IDs using a snapshot of the OpenAlex repository (or via an API call). The IDs, along with the pre-captured structured fields (Title, Abstract, and Scientific Discipline), are passed into the OpenAlex model. The resulting list of Topics is then sent back to the applicant for verification in real time.

**Figure 7:**
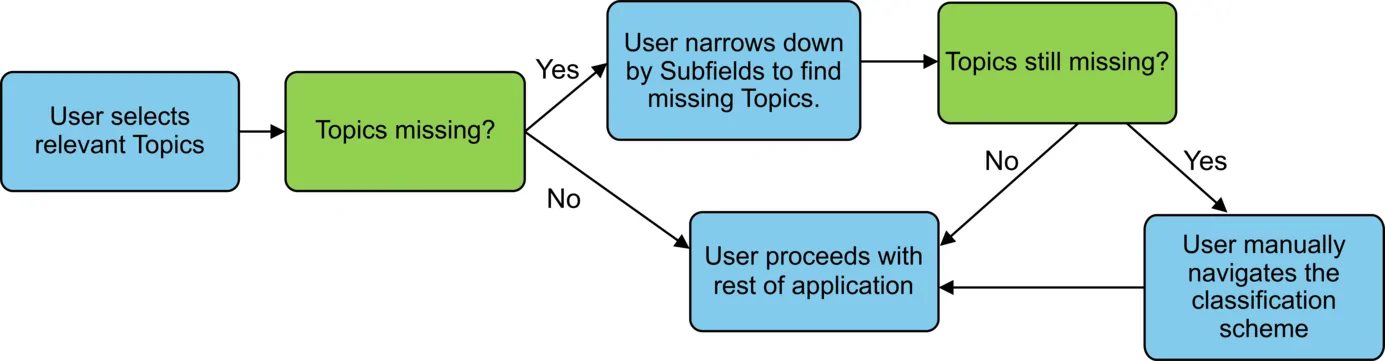
A flowchart of the verification process, representing a guided discovery workflow with successive refinement. The process starts with the predicted Topics, transitions to Subfield filtering in the event of verification gaps, and terminates in a manual hierarchy navigation to ensure 100% classification coverage.

Referring to Fig. 6 ▸, the beam-time applicant, or User, submits their proposal documents along with other necessary information on the User Portal, and the relevant data are extracted and processed to be fed into the OpenAlex model. The predicted Topics will then be displayed to the applicant to be verified in real time. If specific Topics are missing, the applicant can use the predicted Subfields as filters to locate them; this approach is validated by our findings in Section 4.2.1. Should this fail, the User can also manually navigate the classification scheme, starting from the Domains and Fields (which are small enough in numbers), to find the missing Topics. This hierarchical filtering approach, illustrated in Fig. 7 ▸, provides a guided discovery process with a reliable manual fallback.

A key consideration is the expected latency for the live service, specifically the GROBID parsing and the model inferencing. GROBID took 52.39 s for a test run of 10 experiment report PDFs on a MacBook Pro (2023 ▸) with an Apple M3 Pro chip, 18 GB of memory, and no explicit parallel processing setup. This translates to a throughput of 5.24 s per document. Model inferencing performed on the 5384 proposals took about 16 min on the same MacBook Pro setup. This is equivalent to roughly 0.178 s per proposal on average. An actual live service would presumably have access to more advanced hardware, including powerful GPUs. Hence, the expected throughput in a live service setting can reasonably be estimated to be no more than a few seconds per proposal.

In the short term, the live service would fulfil the aim of enhancing metadata granularity of experiment proposals. In the long-term, the system would provide user-validated data that can be used to refine the current model or to train a completely new model that specializes in synchrotron-related technical documents, which could address the challenge of retrospective classification of past proposals.

Furthermore, the live service would also provide another source of feedback for the OpenAlex team, which can be used to further refine the classification scheme and the model architecture. For example, users of the live service can identify gaps in the classification scheme, which can be used to inform future updates and revisions.

### 5.2. Future of OpenAlex within the synchrotron community

The inclusion of proposals and experiment reports into bibliographic databases like OpenAlex is necessary to establish a complete record of the research lifecycle, enabling the scientific community to trace the evolution of a research activity from its foundational concept and experimental design (the proposal and report) to the subsequent data and publications. This would greatly improve research transparency and understanding. Furthermore, a centralized repository that includes proposals and reports unlocks significant data mining opportunities, allowing researchers and funding bodies to track emerging research trends and methodological shifts long before they appear in formal publications. Crucially, it would also provide new ways to evaluate the scientific impact and return on investment of facility time (especially relevant since the proposals can be considered a ‘grant-in-kind’ from the perspective of the facility), by quantitatively linking the initial funding and resources allocated to the final scholarly output.

Currently, ESRF experiment proposals are indexed in OpenAlex as ‘Awards’, a designation that reflects their status as ‘grants-in-kind’. Most of these data are sourced via Crossref, which indexes publications and captures associated award metadata. However, coverage is inconsistent across journals and is largely limited to ESRF. In addition, the ingested metadata are limited to a simple ID, omitting critical fields such as the title, abstract, and author list. Access to the full text of the proposals is further restricted by initial embargoes to protect researcher intellectual property. Finally, although experiment reports could theoretically be ingested as OpenAlex Works (as a ‘Report’ type with the facility, such as ESRF, as the ‘Funder’), they remain completely absent.

Evidently, the full inclusion of proposals and experiment reports into bibliographic databases is a challenge that is hindered by both the facilities’ internal policies and the lower-profile nature of these scholarly outputs among the academic community.

Momentum is building towards addressing these challenges, with various key stakeholders such as Wellcome and the French government endorsing and adopting the OpenAlex ecosystem. This work contributes to this collective effort by validating the alignment of synchrotron data infrastructures with this ecosystem. As OpenAlex continues to refine the classification scheme and the planned live service improves with the increase in User-verified datapoints, OpenAlex will continue to gain prominence within the synchrotron community, and, with it, the proposals and experiment reports among the wider scientific community.

## Figures and Tables

**Figure 8:**
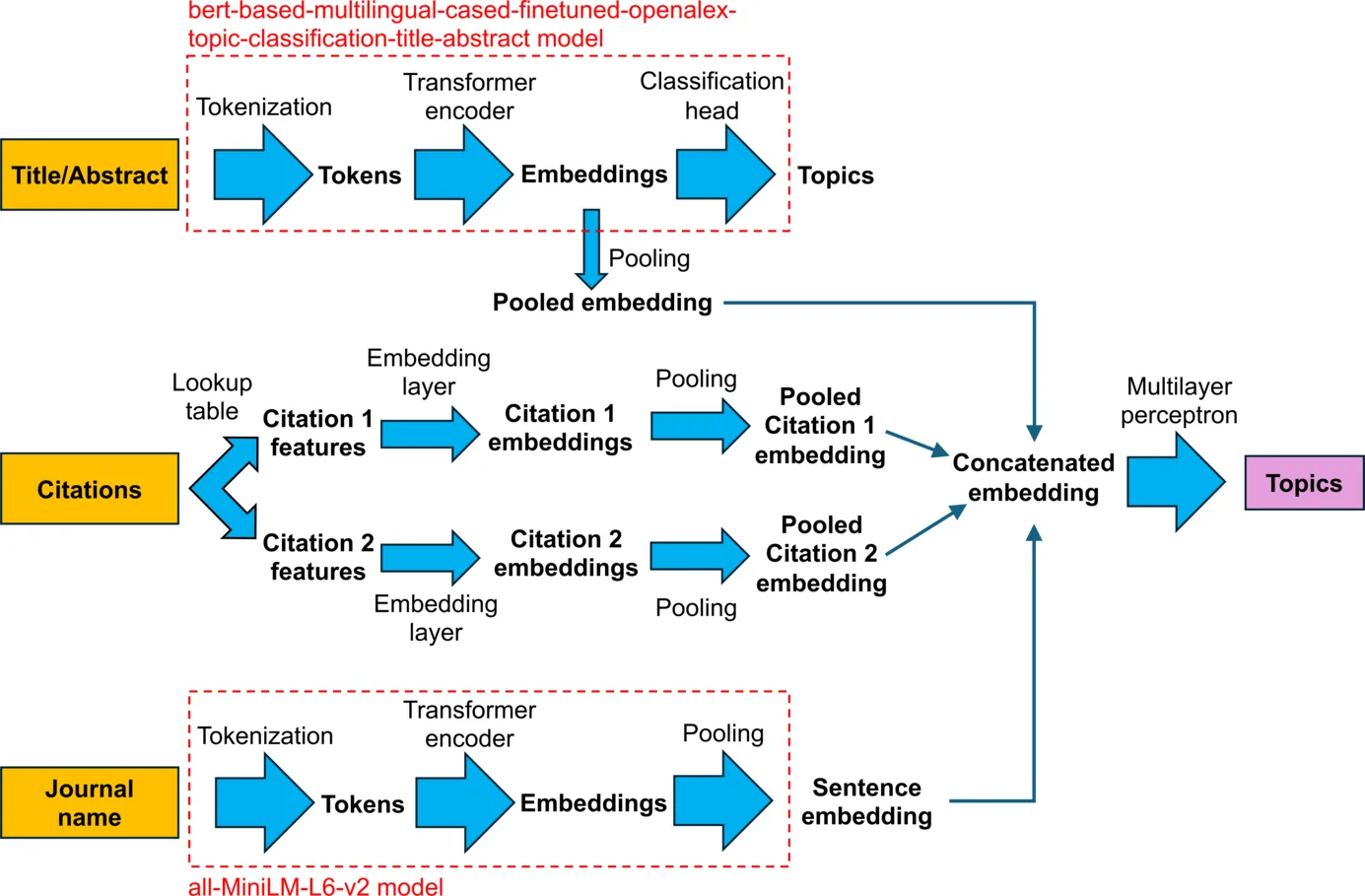
The full OpenAlex topic classification model. Title, abstract, and journal name are processed by separate language models. The citations are first mapped to two lists of integers, before being passed into an embedding layer. All these outputs are then pooled and passed into the multilayer perceptron.

**Figure 9:**
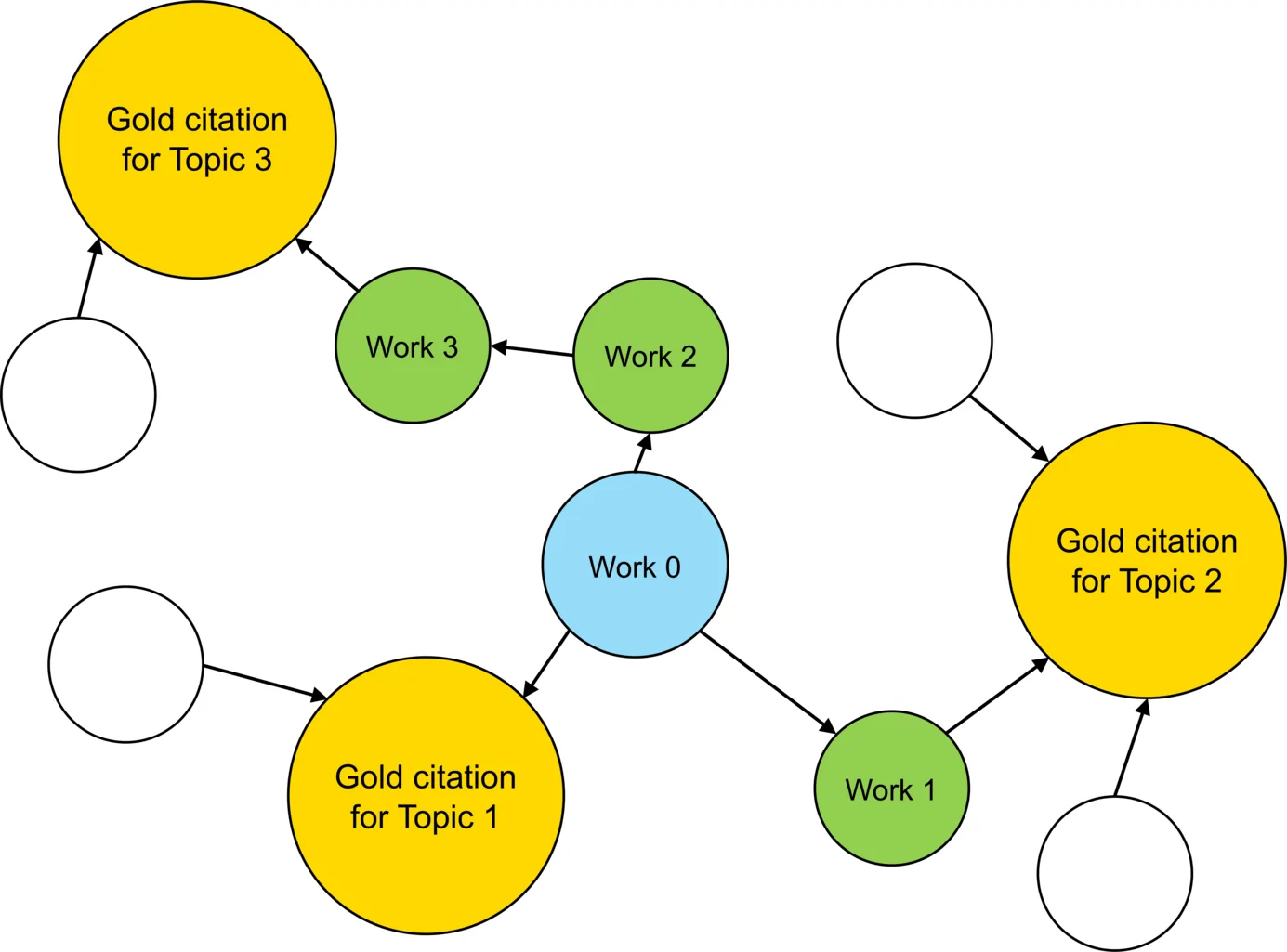
A toy example of the citation graph, adapted from OpenAlex (2024*a* ▸). The large golden circles denote Works that are gold citations for specific topics. The remaining circles represent generic non-gold citation Works and serve to illustrate how frequently the gold citations are referenced. In this example, the blue circle corresponds to Work 0, which is the document being classified. Work 0 has a ‘Citation 1’ feature, which is Topic 1 as Work 0 directly cites the gold citation associated with that Topic. The ‘Citation 2’ feature of Work 0 is Topic 2, as Work 0 cites Work 1, which in turn cites a gold citation of Topic 2. Because the citation path from Work 0 to the gold citation of Topic 2 spans two graph edges, Topic 2 is a second-order citation feature of Work 0. Topic 3 is irrelevant in this case, as Work 0 is more than 2 edges away.

**Figure 10:**
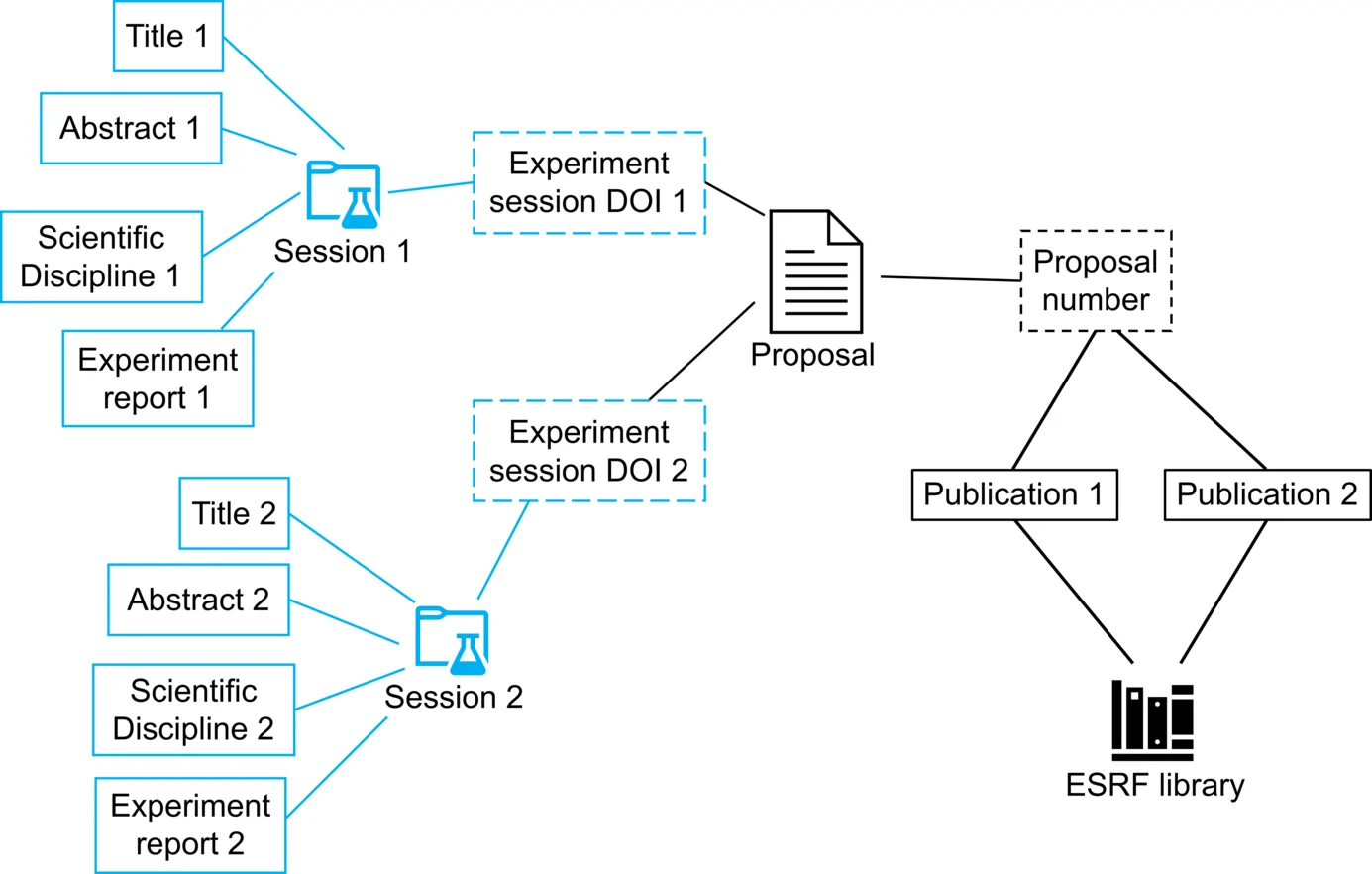
The data ecosystem linking ESRF research outputs. This figure illustrates the web of information, connecting ESRF proposals to experiment reports (via experiment session DOIs), and proposals to publications (via proposal numbers), which are indexed by the ESRF library. Each experiment session is tagged with metadata: Title, Abstract, Scientific Discipline, and the URL to the associated experiment report. The dashed squares represent unique identifiers that link the various elements.

**Table 1:** Example of an OpenAlex Topic with its hierarchy and associated metadata

Hierarchy	
Domain	Health Sciences
Field	Health Professions
Subfield	Health Information Management

Topic metadata	
Display Name	Artificial Intelligence in Healthcare
ID	https://openalex.org/T11396
Description	This cluster of papers focuses on the application of machine learning, big data analytics, and data mining techniques in healthcare and medicine. It covers topics such as medical diagnosis, classification models, heart disease prediction, and the use of support vector machines and logistic regression. The cluster emphasizes the potential of machine learning in improving healthcare outcomes through advanced data analysis.
Keywords	Machine Learning, Healthcare, Big Data Analytics, Medical Diagnosis, Classification Models, Heart Disease Prediction, Data Mining, Support Vector Machine, Logistic Regression, Feature Selection

**Table 2:** Reviewer-to-Scientific Discipline Assignments

	ESRF Scientific Disciplines											
	CH	ES	EV	HC	HG	LS	MA	MD	ME	MI	MX	SC
Reviewer 1	•						•					
Reviewer 2	•											
Reviewer 3		•	•	•				•	•		•	
Reviewer 4			•	•	•		•		•	•		
Reviewer 5						•		•			•	
Reviewer 6	•			•							•	
Reviewer 7						•					•	
Reviewer 8						•		•			•	
Reviewer 9												•
Reviewer 10		•										
Reviewer 11				•			•					
Reviewer 12				•			•			•		
Reviewer 13					•				•			
Reviewer 14	•						•			•		
Reviewer 15												•
Reviewer 16		•										
Reviewer 17			•									
Reviewer 18												•

**Table 3:** Inter-rater agreement metrics by Scientific Discipline

Scientific Discipline	Fleiss’ kappa	Randolph’s kappa	Krippendorff’s alpha
Chemistry	0.463	0.547	0.574
Hard Condensed Matter Science	0.511	0.652	0.616
Applied Material Science	0.391	0.514	0.497
Soft Condensed Matter Science	0.418	0.443	0.614
Others	0.381	0.440	0.513
**All**	**0.445**	**0.525**	**0.572**

**Table 4:** Number/proportion of proposals with at least *n* Topics with mean reviewer score *s* ≥ τ, where τ is the threshold for ‘correctness’

Scientific Discipline	≥ 1 Topic	≥ 2 Topics	3 Topics	Total
(*a*) τ = 0.667				
Chemistry	38 (88.4%)	22 (51.2%)	6 (14.0%)	43
Hard Condensed Matter Science	44 (97.8%)	36 (80.0%)	12 (26.7%)	45
Applied Material Science	46 (97.9%)	31 (66.0%)	11 (23.4%)	47
Soft Condensed Matter Science	26 (89.7%)	16 (55.2%)	1 (3.45%)	29
Others	38 (84.4%)	21 (46.7%)	3 (6.67%)	45
**All**	**192 (91.9%)**	**126 (60.3%)**	**33 (15.9%)**	**209**

(*b*) τ = 0.833				
Chemistry	34 (79.1%)	13 (30.2%)	1 (2.33%)	43
Hard Condensed Matter Science	43 (95.6%)	32 (71.1%)	6 (13.3%)	45
Applied Material Science	41 (87.2%)	21 (44.7%)	6 (12.8%)	47
Soft Condensed Matter Science	23 (79.3%)	10 (34.5%)	1 (3.45%)	29
Others	32 (71.1%)	10 (22.2%)	1 (2.22%)	45
**All**	**173 (82.8%)**	**86 (41.1%)**	**15 (7.18%)**	**209**

(*c*) τ = 1				
Chemistry	31 (72.1%)	10 (23.3%)	0 (0.00%)	43
Hard Condensed Matter Science	38 (84.4%)	29 (64.4%)	4 (8.89%)	45
Applied Material Science	39 (83.0%)	15 (31.9%)	1 (2.13%)	47
Soft Condensed Matter Science	19 (65.5%)	6 (20.7%)	0 (0.00%)	29
Others	28 (62.2%)	4 (8.89%)	0 (0.00%)	45
**All**	**155 (74.2%)**	**64 (30.6%)**	**5 (2.39%)**	**209**

**Table 5:** Ablation study of each feature for the 209 experiment proposals

Excluded feature	Number of proposals with different outputs
Title	122 (58.4%)
Abstract	184 (88.0%)
Citations	129 (61.7%)
Scientific Discipline	10 (4.78%)

**Table 6:** List of proposals with different outputs after excluding Scientific Discipline The ‘Topic ID and label’ column represents the Topics that were dropped by the model when the Scientific Discipline was excluded.

Proposal number	Topic ID and label	Mean score
CH-5953	11279: Lanthanide and Transition Metal Complexes	0.667
CH-6158	10152: Animal Nutrition and Physiology	0.5
ES-1266	11733: X-ray Spectroscopy and Fluorescence Analysis	0.833
MA-4760	13104: Chemical and Physical Properties of Materials	1
MX-2415	12104: Protein Tyrosine Phosphatases	0.167
MX-2443	11162: Enzyme Structure and Function	0.667
SC-5103	10343: Hydrogels: synthesis, properties, applications	1
SC-5228	10666: Photonic Crystals and Applications	1
SC-5319	10729: Electrospun Nanofibers in Biomedical Applications	0.167
SC-5384	11799: Adhesion, Friction, and Surface Interactions	0.667

## Data Availability

The code and datasets can be found on the following GitHub repository: https://github.com/terencetan-c/esrf-proposals-topic-classification.

## Appendix A Model architecture

The OpenAlex topic classification model comprises a sentence embedding model (Sentence Transformers, 2021 ▸), a title/abstract classifier model (OpenAlex, 2024*c* ▸), a lookup table, and a feedforward neural network with three hidden layers; the latter is also known as a multilayer perceptron (MLP). The sentence embedding model creates numerical representations of the journal names, called embeddings. The title/abstract model was built by fine-tuning the BERT multilingual base model (cased) (Devlin *et al.*, 2019 ▸) on title/abstract to perform topic classification based only on the titles and abstracts of Works. The MLP has three hidden layers using the Rectified Linear Unit (ReLU) activation function, followed by an output layer with a sigmoid activation function. Using these components, the model processes each input in the following way (refer to Fig. 8 ▸ for a visual representation):

(i) **Title/Abstract**. The concatenated title and abstract are passed into the title/abstract model, where they are first broken down into smaller subunits known as tokens. These could be individual words, punctuation, and/or subwords. These tokens are then passed through the transformer encoder to give contextualized vector representations known as embeddings. These embeddings would normally go through the title/abstract model’s classification head to give topic predictions. However, in the case of the full model, the embeddings are pooled, converting the sequence of embeddings into a compressed, lower-dimensional embedding without losing too much of the contextual information.

(ii) **Journal name**. Similarly, the sentence embedding model first tokenizes the journal name, then encodes the resulting tokens, before pooling the resulting sequence of embeddings to give a single sentence embedding that numerically represents the entire journal name.

(iii) **Citations**. The full model relies on what the OpenAlex team refers to as gold citations to process the citations. These gold citations represent the highest cited works for each Topic, and their prominence is exclusive to that Topic. Each Topic has about 10–75 gold citations, adding up to a total of approximately 120000 gold citations. The list of citations are filtered and grouped into two categories: Citation 1 (gold citations) and Citation 2 (Works that directly cite a gold citation). The lookup table first maps these Citation 1 and Citation 2 entries to their respective Topics, and subsequently maps these Topics to integers. This produces two distinct lists of integers, called Citation 1 features and Citation 2 features, which are then passed into an embedding layer. The resulting embeddings are then pooled and ready for input to the MLP. Fig. 9 ▸ shows a toy example of this process in terms of a network graph.

The four input features, the pooled embedding of the title and abstract, the pooled embedding of the journal name, the pooled Citation 1 embedding, and the pooled Citation 2 embedding, are passed into the MLP to produce the output vector , as explained in Section 2.4.

## Appendix B Getting the dataset

ESRF has made great progress towards the FAIRification of their scholarly outputs, with the Data Portal (https://data.esrf.fr) containing experiment data, with the Joint ILL-ESRF Library (https://epn-library.esrf.fr/) that indexes publications associated with ESRF and Institut Laue-Langevin (ILL) experiments, and with the ICAT experiment metadata catalogue (Collaboration, 2014 ▸). The latter serves as the central, secure repository for structured information about experiment reports, datasets, and users, and can be accessed through a web browser or programmatically via an API (https://icatplus.esrf.fr).

The various unique identifiers, both global (DOIs) and local (ESRF-designated proposal numbers), enable traversal between and within the ESRF data ecosystem and the OpenAlex repository (see Fig. 10 ▸). This facilitated the collection of the required data associated with each proposal.

## References

[bb1] Australian Bureau of Statistics (2020). Australian and New Zealand Standard Research Classification (ANZSRC) https://www.abs.gov. au/statistics/classifications/australian-and-new-zealand-standard-research-classification-anzsrc/latest-release.

[bb2] Brugger, J. (2025). *The nature of uranium in shales* [dataset] https://doi.esrf.fr/10.15151/ESRF-ES-649328472.

[bb3] Castro, S. (2017). *Fast Krippendorff: Fast computation of Krippendorff’s alpha agreement measure*https://github.com/pln-fing-udelar/fast-krippendorff.

[bb4] Cau, A., Beyrand, V., Voeten, D. F. A. E., Fernandez, V., Tafforeau, P., Stein, K., Barsbold, R., Tsogtbaatar, K., Currie, P. J. & Godefroit, P. (2017). *Nature* **552**, 395–399. 10.1038/nature24679 29211712

[bb5] Chang, J., Von Arx, K. M. & Qisi Wang (2024). *Probing the low-energy orbital texture of mott insulating ca2ruo4* [dataset] https://doi.esrf.fr/10.15151/ESRF-ES-514143370.

[bb6] Ciani, M., Lepore, G. O. & Margheri, S. (2026). *Chemical and structural characterization of metal-organic materials obtained through heavy metal biosorption by exopolysaccharide-producing* [dataset] European Synchrotron Radiation Facility https://doi.esrf.fr/10.15151/ESRF-ES-1234126108.

[bb7] Cohen, J. (1960). *Educ. Psychol. Meas.* **20**, 37–46.

[bb8] Collaboration, T. I. C. A. T. (2014). *The ICAT Project.**The ICAT Collaboration.*https://doi.org/10.5286/SOFTWARE/ICAT.

[bb9] Devlin, J., Chang, M.-W., Lee, K. & Toutanova, K. (2019). *Proceedings of the 2019 Conference of the North American Chapter of the Association for Computational Linguistics: Human Language Technologies*, Volume 1 (Long and Short Papers), edited by J. Burstein, C. Doran & T. Solorio, pp. 4171–4186.

[bb10] Dewaele, A., Henry, L., Faure, P. & Freville, R. (2025). *Static phase diagram of tin above 1 mbar* [dataset]. https://doi.esrf.fr/10.15151/ESRF-ES-974856617.

[bb11] Douangamath, A., Fearon, D., Gehrtz, P., Krojer, T., Lukacik, P., Owen, C. D., Resnick, E., Strain-Damerell, C., Aimon, A., Ábrányi-Balogh, P., Brandão-Neto, J., Carbery, A., Davison, G., Dias, A., Downes, T. D., Dunnett, L., Fairhead, M., Firth, J. D., Jones, S. P., Keeley, A., Keserü, G. M., Klein, H. F., Martin, M. P., Noble, M. E. M., O’Brien, P., Powell, A., Reddi, R. N., Skyner, R., Snee, M., Waring, M. J., Wild, C., London, N., von Delft, F. & Walsh, M. A. (2020). *Nat. Commun.* **11**, 5047. 10.1038/s41467-020-18709-w PMC7542442 33028810

[bb12] ESRF (2020). *ESRF Highlights 2020*https://www.esrf.fr/home/UsersAndScience/Publications/Highlights/esrf-highlights-2020.html.

[bb13] ESRF (2021). *ESRF Highlights 2021*https://www.esrf.fr/home/UsersAndScience/Publications/Highlights/esrf-highlights-2021.html.

[bb14] ESRF (2022). *ESRF Highlights 2022*https://www.esrf.fr/home/UsersAndScience/Publications/Highlights/esrf-highlights-2022.html.

[bb15] ESRF (2023). *ESRF Highlights 2023*https://www.esrf.fr/home/UsersAndScience/Publications/Highlights/esrf-highlights-2023.html.

[bb16] ESRF (2024). *ESRF Highlights 2024*https://www.esrf.fr/home/UsersAndScience/Publications/Highlights/esrf-highlights-2024.html.

[bb17] Fenner, M. (2025). *Rogue scholar is improving subject classification* (version 2) https://dx.doi.org/10.53731/4pr0j-7pq24.

[bb18] Fleiss, J. L. (1971). *Psychol. Bull.* **76**, 378–382.

[bb19] Gasparyan, A. Y., Ayvazyan, L. & Kitas, G. D. (2013). *J. Kor. Med. Sci.* **28**, 1270–1275. 10.3346/jkms.2013.28.9.1270 PMC3763098 24015029

[bb20] GROBID (2008–2025). *Grobid*https://github.com/kermitt2/grobid.

[bb21] Krippendorff, K. (2019). *Content analysis: an introduction to its methodology*, 4th ed. Los Angeles: SAGE Publications.

[bb22] McHugh, M. L. (2012). *Biochem. Med.* **22**, 276–282. PMC3900052 23092060

[bb23] Meuschke, N., Jagdale, A., Spinde, T., Mitrović, J. & Gipp, B. (2023). *Information for a Better World: Normality, Virtuality, Physicality, Inclusivity*, Vol. 13972 of *Lecture Notes in Computer Science (LNCS)*, pp. 383–405. Cham: Springer Nature Switzerland.

[bb24] Ministry of Higher Education, Research and Innovation (2021). *Second French Plan for Open Science*https://www.ouvrirlascience.fr/wp-content/uploads/2021/10/Second_French_Plan-for-Open-Science_web.pdf.

[bb25] Ministry of Higher Education, Research and Innovation (2024). *French Ministry of Higher Education and Research partners with OpenAlex to develop a fully open bibliographic tool*https://www.ouvrirlascience.fr/french-ministry-of-higher-education-and-research-partners-with-openalex-to-develop-a-fully-open-bibliographic-tool/.

[bb27] OpenAlex (2024*a*). *Openalex: End-to-end process for topic classification*https://docs.google.com/document/d/1bDopkhuGieQ4F8gGNj7sEc8WSE8mvLZS/edit#heading=h.5w2tb5fcg77r.

[bb28] OpenAlex (2024*b*). *OpenAlex topic classification v1 model artifacts and training data*https://doi.org/10.5281/zenodo.10568401.

[bb26] OpenAlex (2024*c*). *bert-base-multilingual-cased-finetuned-openalex-topic-classification-title-abstract*https://huggingface.co/OpenAlex/bert-base-multilingual-cased-finetuned-openalex-topic-classification-title-abstract.

[bb29] Porter, S. J., Hawizy, L. & Hook, D. W. (2023). *Quant. Sci. Stud.* **4**, 127–143.

[bb30] Priem, J. (2025). *OpenAlex blog*https://blog.openalex.org/were-rebuilding-openalex-while-its-running-heres-whats-changing/.

[bb31] Priem, J., Piwowar, H. & Orr, R. (2022). *Openalex: A fully open index of scholarly works, authors, venues, institutions, and concepts*https://arxiv.org/abs/2205.01833.

[bb32] Randolph, J. J. (2005). *Free-Marginal Multirater Kappa (multirater K[free]): An Alternative to Fleiss’ Fixed-Marginal Multirater Kappa.*

[bb33] Seabold, S. & Perktold, J. (2010). *9th Python in Science Conference*, 28 June–3 July 2010, Austin, TX, USA, pp. 57-61.

[bb34] Sentence Transformers (2021). *all-minilm-l6-v2*, https://huggingface.co/sentence-transformers/all-MiniLM-L6-v2.

[bb35] Siebenschuh, C., Hippe, K., Gokdemir, O., Brace, A., Khan, A., Hossain, K., Babuji, Y., Chia, N., Vishwanath, V., Stevens, R., Ramanathan, A., Foster, I. & Underwood, R. (2025). *Adaparse: An adaptive parallel pdf parsing and resource scaling engine*https://arxiv.org/abs/2505.01435.

[bb36] Simard, M.-A., Basson, I., Hare, M., Lariviere, V. & Mongeon, P. (2024). *The open access coverage of openalex, scopus and web of science*https://arxiv.org/abs/2404.01985.

[bb37] Singh, V. K., Singh, P., Karmakar, M., Leta, J. & Mayr, P. (2021). *Scientometrics* **126**, 5113–5142.

[bb38] Tan, T. (2024–2025). *Topic classification of esrf experiment proposals*https://github.com/terencetan-c/esrf-proposals-topic-classification.

[bb39] Traag, V. A., Waltman, L. & van Eck, N. J. (2019). *Sci. Rep.* **9**, 5233. 10.1038/s41598-019-41695-z PMC6435756 30914743

[bb40] Van Eck, N. J. (2024). *Classification of research publications based on data from openalex*https://zenodo.org/doi/10.5281/zenodo.10560275.

[bb41] Van Eck, N. J., Visser, M. & Waltman, L. (2024). *Opening up the CWTS Leiden Ranking: Toward a decentralized and open model for data curation*https://doi.org/10.59350/fn8b7-69107.

[bb42] Van Eck, N. J. & Waltman, L. (2024). *An open approach for classifying research publications*https://doi.org/10.59350/qc0px-76778.

[bb43] Waltman, L., Boyack, K. W., Colavizza, G. & van Eck, N. J. (2020). *Quant. Sci. Stud.* **1**, 691–713.

[bb44] Wellcome (2025). *Wellcome announces suite of open research measures*https://wellcome.org/insights/articles/wellcome-announces-suite-open-research-measures.

[bb45] Wilkinson, M. D., Dumontier, M., Aalbersberg, I. J., Appleton, G., Axton, M., Baak, A., Blomberg, N., Boiten, J.-W., da Silva Santos, L. B., Bourne, P. E., Bouwman, J., Brookes, A. J., Clark, T., Crosas, M., Dillo, I., Dumon, O., Edmunds, S., Evelo, C. T., Finkers, R., Gonzalez-Beltran, A., Gray, A. J., Groth, P., Goble, C., Grethe, J. S., Heringa, J., ’t Hoen, P. A., Hooft, R., Kuhn, T., Kok, R., Kok, J., Lusher, S. J., Martone, M. E., Mons, A., Packer, A. L., Persson, B., Rocca-Serra, P., Roos, M., van Schaik, R., Sansone, S.-A., Schultes, E., Sengstag, T., Slater, T., Strawn, G., Swertz, M. A., Thompson, M., van der Lei, J., van Mulligen, E., Velterop, J., Waagmeester, A., Wittenburg, P., Wolstencroft, K., Zhao, J. & Mons, B. (2016). *Sci. Data* **3**, 160018.

